# A systematic review of scabies transmission models and data to evaluate the cost-effectiveness of scabies interventions

**DOI:** 10.1371/journal.pntd.0007182

**Published:** 2019-03-08

**Authors:** Naomi van der Linden, Kees van Gool, Karen Gardner, Helen Dickinson, Jason Agostino, David G. Regan, Michelle Dowden, Rosalie Viney

**Affiliations:** 1 Centre for Health Economics Research and Evaluation, University of Technology Sydney, Sydney, Australia; 2 Public Service Research Group, School of Business UNSW Canberra, Canberra, Australia; 3 Academic Unit of General Practice, Australian National University, Canberra, Australia; 4 The Kirby Institute, UNSW Sydney, Sydney, Australia; 5 One Disease, Darwin, Australia; Institute for Disease Modeling, UNITED STATES

## Abstract

**Background:**

Scabies is a common dermatological condition, affecting more than 130 million people at any time. To evaluate and/or predict the effectiveness and cost-effectiveness of scabies interventions, disease transmission modelling can be used.

**Objective:**

To review published scabies models and data to inform the design of a comprehensive scabies transmission modelling framework to evaluate the cost-effectiveness of scabies interventions.

**Methods:**

Systematic literature search in PubMed, Medline, Embase, CINAHL, and the Cochrane Library identified scabies studies published since the year 2000. Selected papers included modelling studies and studies on the life cycle of scabies mites, patient quality of life and resource use. Reference lists of reviews were used to identify any papers missed through the search strategy. Strengths and limitations of identified scabies models were evaluated and used to design a modelling framework. Potential model inputs were identified and discussed.

**Findings:**

Four scabies models were published: a Markov decision tree, two compartmental models, and an agent-based, network-dependent Monte Carlo model. None of the models specifically addressed crusted scabies, which is associated with high morbidity, mortality, and increased transmission. There is a lack of reliable, comprehensive information about scabies biology and the impact this disease has on patients and society.

**Discussion:**

Clinicians and health economists working in the field of scabies are encouraged to use the current review to inform disease transmission modelling and economic evaluations on interventions against scabies.

## Author summary

Scabies is a neglected tropical disease affecting more than 130 million people, with major costs on health care systems worldwide. While effective treatments exist, it is unknown which treatment strategies result in the best outcomes against the lowest costs, and to what extent this differs between communities. Health economic modelling can help answer these questions, but has rarely been used in this disease area. This review discusses all available scabies transmission models (n = 4), and uses them to create a new, comprehensive modelling framework. This framework can be used as aid for creating a scabies transmission model, the details of which will be determined by the context (population) and the question being addressed. The current paper also reviews the data that is needed to inform scabies modelling: on scabies biology, quality of life and resource use. Unfortunately, available data is limited and particularly data on crusted scabies (associated with high morbidity and mortality rates) is rare. With this review, we hope to assist researchers and policy makers to predict and/or evaluate the cost-effectiveness of interventions against scabies in their population(s) of interest. To tackle scabies, it is key to use effective treatment strategies in a cost-effective and sustainable way. The models and data described in this review, may help researchers, clinicians and funding bodies to facilitate this.

## Introduction

Scabies is a common dermatological conditions [1], affecting more than 130 million people at any time [2]. It is a neglected disease caused by the mite *Sarcoptes Scabiei* [3]. Scabies often results in severe itching, and in some patients, including those with compromised immunity, it may progress to “crusted scabies” (CS). The fissures associated with scabies provide a portal of entry for bacteria, potentially resulting in secondary infections, sepsis, indirect effects on renal and cardiovascular function, and death due to complications [4]. Secondary bacterial superinfections are uncommon in Western countries [5].

Worldwide, scabies is responsible for 0.07% of the total burden of disease [6]. Compared to its disease burden, scabies research is severely underfunded [7, 8], even though it imposes major costs on healthcare systems [2]. Various countries and organisations have identified scabies control as a public health priority and the World Health Organisation Strategic and Technical Advisory Group for Neglected Tropical Diseases recently recommended that scabies be included in the Neglected Tropical Disease profile in category A [2, 9–11].

Elimination of scabies is difficult, as cured patients often get re-infected. Treatment strategies range from treating individuals and their contacts, to mass drug administration (MDA) strategies [12–19], which involves treating whole communities at once. Drugs include oral ivermectin as well as a range of topical treatment options. It is unknown which (combination of) treatment strategies results in the best health outcomes against the lowest costs, and to what extent this differs between communities. Health-economic modelling may help answer such questions. To determine the cost-effectiveness of interventions against scabies, it is crucial to take into account it’s infectious nature since the extent to which interventions impact transmission will (to a large part) determine their cost-effectiveness. While such disease transmission approaches have successfully been applied to guide interventions against other infectious diseases like Ebola and influenza [20], few attempts have been made to use modelling to aid decision-making about scabies intervention strategies.

Scabies interventions include efforts aimed at access and coordination of services, scabies detection, primary care, acute care, specialised care, social work, follow-up, or combinations of the above. Given the multifaceted nature of interventions required to combat scabies, health-economic evaluation requires a comprehensive modelling approach. For this article, a systematic review of existing scabies models was conducted to inform the development of a proposed modelling framework which can be adjusted to different situations/communities. The proposed modelling framework aims to determine long-term effects of alternative interventions on the incidence, prevalence, quality of life (QoL), resource use and costs associated with scabies and CS. It can be used as aid for creating a scabies transmission model, the details of which will be determined by the context (population) and the question being addressed.

For models to be of use for decision-makers, a range of clinical and economic inputs is required. However, up to now, no systematic overview of evidence-based information on these inputs, including evidence on the biology of scabies, patient QoL and resource use has been published. This systematic literature review fills this gap by providing an overview of published information that can be used to inform scabies modelling in human populations, and improve decision-making about scabies interventions in affected communities.

After discussing the search strategy, this paper will first discuss characteristics of published scabies models and a proposed, comprehensive scabies modelling framework. Secondly, the paper will discuss potential model inputs, consecutively: the life cycle of scabies mites, patient QoL, and resource use associated with scabies and CS.

## Methods

In order to inform our proposed modelling framework design, a systematic literature search was performed on 26 and 27 July 2017, searching the databases PubMed, Medline, Embase, CINAHL, and the Cochrane Library. Search terms related to the disease ("scabies" OR "sarcoptes") were combined with search terms identifying the type of information required ("model" OR “modeling” OR “modelling” OR "transmission" OR "utility" OR “quality of life” OR “economics” OR “economic” OR “cost-effectiveness” OR “cost-utility” OR “cost” OR “cost-of-illness” OR “cost-consequence” OR “cost-consequences” OR “efficacy” OR “effectiveness” or “impact”).

Articles were limited to humans only, had to be published in English and after the year 2000. Studies from before the year 2000 were only included if they were cited in a more recent source (post 2000), confirming their continued relevance. Studies that considered scabies but did not present any models or model inputs on scabies biology, QoL or resource use were excluded. Articles were also excluded when they presented a case study of a single patient or an outbreak of scabies in a single institution, and if they simply provided a discussion of scabies guidelines or protocols in a particular country or institution. Those studies that mentioned scabies as one of a number of diseases/indications/causes/comorbidities were also excluded. Reference lists of reviews were used to identify any papers missed through the search strategy.

Data extractions were performed by the primary author, per topic area: (1) models, (2) biology of scabies, (3) patient QoL and (4) resource use. Data items were not predefined, meaning that all data on the various topic areas (e.g. QoL) was included and presented, independent of reported outcome measures (e.g. which type of QoL questionnaire was used). After the data was extracted per topic area, it was evaluated which data per topic addressed simple scabies, CS, and which were based on populations including both simple scabies and CS patients. Results were not meta-analysed.

The validity and reliability of disease models and inputs is highly dependent on the research question they try to answer, the population of interest, and how the models are informed/how the inputs are used. Therefore, this review did not include a quantitative risk of bias assessment, but a qualitative description of the limitations of the various models and input parameters. All scabies models were evaluated on their main characteristics, and strengths and limitations were identified. Strengths of the various models were combined to design a new, proposed modelling framework. Relevant data from papers on the biology of scabies, patient QoL and resource use was extracted and described.

A review protocol has not been published. A completed PRISMA checklist is provided as supplementary material.

## Results

**Fig 1:**
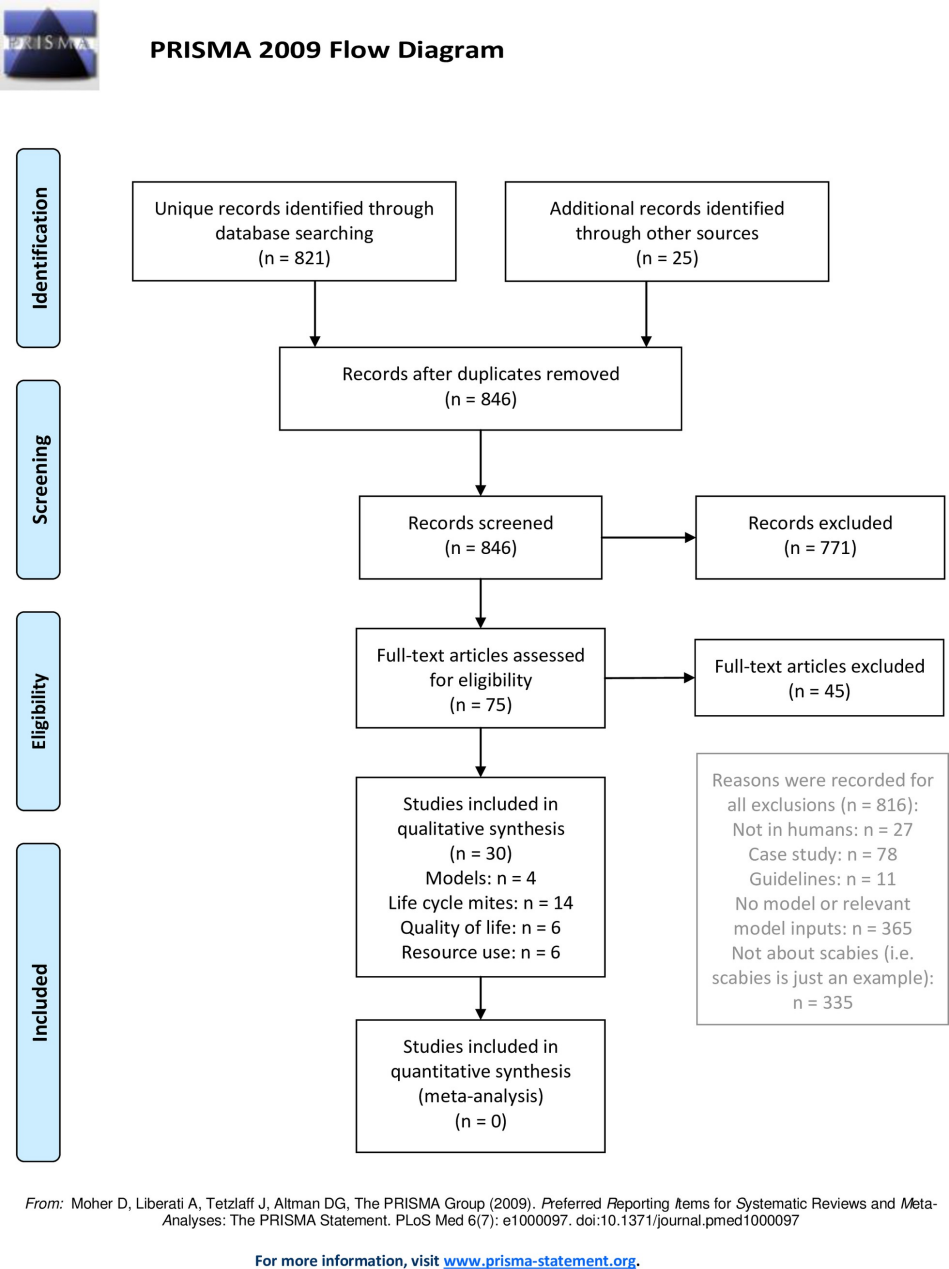
Systematic review flow diagram.

The literature search identified 821 articles, 30 of which were included. Fig 1 provides information on article selection, reasons for exclusion, and categorisation of included articles.

### Published scabies models

Four scabies models were identified from the literature (Table 1). One model was a Markov decision tree, two were compartmental models, and one was an agent-based, network-dependent Monte Carlo model (see Table 2 for a description of these model types). None of the models identified specifically addressed CS.

**Table 1:** Published scabies models.

	[21]	[22]	[23]	[24]
**Model type**	Markov decision tree.	Agent-based, network-dependent Monte Carlo model.	Deterministic, compartmental (SIR) model.	Compartmental (SIR) model.
**Software**	NR	Mathematica 7.0	MATLAB	MATLAB
**Indication**	Scabies	Scabies	Scabies	Scabies
**Aims**	-Determine the cost-effectiveness of six alternative treatment regimens	-Insight into scabies dynamics -Determine effects of treatment strategies	-Show endemic equilibria. -Determine whether the current treatment regime is enough to control the disease, or whether a vaccine is needed.	-Explore the impact of MDA treatment strategies. -Capture differences between ovicidal and non-ovicidal treatments.
**Perspective**	Patient perspective	Community perspective	Community perspective	Community perspective
**Lead-in/relaxation time**^**1**^	Not relevant.	12 months.	NR	NR
**Probability of infection depends on**	Not considered.	-Transmissibility parameter. -Number of first-degree links that are positive for scabies. -Age. -Genetic susceptibility. -Importation likelihood.	-Contact rate and infectiousness. -Vaccination rate, protective effect of vaccine, and waning rate of vaccine. -Protective effect of prior infection. -Recovery rate.	-Life cycle of the mites. -Contact rate and infectiousness. -Relative susceptibility to subsequent infections, and relative infectiousness of those with subsequent infections. -MDA coverage, background treatment rate and efficacy.
**Sources of key parameters**	Clinical trial	Calibrated based on published prevalence and incidence rates	Published data (vaccination and vaccine waning rates based on tuberculosis vaccines), and Central Statistical Office of Zimbabwe	Published data
**Mixing**	Not taken into account.	A range of small-world network architectures was tested.	Not modelled (homogeneous mixing implicitly assumed).	Not modelled (homogeneous mixing implicitly assumed).
**Network/population size**	Not taken into account.	200 (100, 500 and 1,000 tested)	1,000	2,000
**Scenarios**	None.	-Treating index cases and all first-degree contacts. -Treating index cases only.	-No intervention. -Vaccination as only intervention. -Scabies treatment as only intervention. -Vaccination and scabies treatment are combined.	-Varying intervention intervals. -Varying number of successive interventions. -Non-ovicidal versus ovicidal treatment. -Varying MDA coverage.
**Analysis**	Cohort-based cost-effectiveness analysis.	Monte Carlo approach and mean-field approximation.^2^	Descartes’ rule of signs and numerical simulations.	Simulation using the Gillespie algorithm and mean-field approximation. Optimisation.
**Outcome measures**	Cost-effectiveness based on total cost of medicines at the end of two weeks and cure rate	Prevalence rates	Prevalence rates	Prevalence of infection, proportion of the population with eggs, probability of extinction.
**Conclusions**	-The cheapest regimen is to give benzyl benzoate for 2 weeks, and then treat uncured pts with ivermectin for the next 2 weeks. Treating patients with 2 weeks of ivermectin only gives the fastest results in half the duration with double the cost of the above regimen. A third regimen (1 week of benzyl benzoate, and then 2 weeks of ivermectin for uncured pts) is also considered cost-effective, providing 100% cure in 3 weeks.	-Scabies burden is adversely affected by increases in average network degree, prominent network clustering, and greater transmissibility. -A community-specific model allows for the determination of an effective treatment protocol that can satisfy any pre-defined target prevalence. -Frequent, low-density treatment protocols are most advantageous. - In the absence of an effective vaccination, and with scabies continually imported to communities from non-local contacts, eradication is impossible and open-ended treatment regimens are needed.	-Vaccination can partially reduce scabies infection, but treatment alone can already reduce scabies levels. -Vaccination combined with treatment is the most effective way to control scabies, but a vaccine does not yet exist. -Disease eradication would only be possible if the reproduction number would be less than unity.	-Even with 100% coverage and efficacy for a non-ovicidal treatment, rebound of infection levels is inevitable. -In an MDA with non-ovicidal treatment, the intervention interval should be ˜2 weeks.

Abbreviations: MDA, mass drug administration; NR, not reported; SIR, susceptible-infectious-recovered.

^1^ This is the time it takes before steady-state prevalence rates are reached.

^2^ A mean field approach here refers to the deterministic (compartmental) implantation of the stochastic model.

**Table 2:** Description of the various model types.

Model type	Description
Markov decision tree	A Markov decision tree is based on a set of health states and transition probabilities to move from one health state to another. Results can be analysed by simulating state membership of a cohort of patients over time.
Compartmental model	In a compartmental model, the population is subdivided into “compartments” that represent a certain health state, e.g. “susceptible (S)”, “infected (I)”, or “recovered (R)”. The interaction between these compartments can be determined based on differential equations or stochastic modelling methods.
Agent-based Monte Carlo model.	An agent-based model considers the effect of the actions and interactions between individual parts (“agents”, e.g. people) on the system as a whole (e.g. transmission of an infection in a community). “Monte Carlo” refers to an algorithm using random sampling to obtain results.

Bachewar et al. [21] published the Markov decision tree as part of a randomised clinical trial comparing three alternative treatment regimens. As opposed to the other models, this model does not consider scabies epidemiology, transmission or population dynamics. A Markov decision tree is provided, which is used to calculate the cost-effectiveness of alternative treatments, using efficacy data from the trial. The model serves as a mechanism to calculate and compare costs for a range of interventions, without considering the biology or transmission of scabies. This limits its use compared to the other identified models.

Gilmore [22] published an agent-based Monte Carlo model, using a variety of small-world network architectures to gain insight into scabies dynamics and the effect of alternative treatment strategies. This study focused on childhood scabies and found that in the absence of an effective vaccine, and with scabies continually imported to communities from non-local contacts, eradication is impossible and open-ended treatment regimens are required. A crucial advantage of Gilmore’s model is that it allows for non-random mixing patterns [25], since it is likely that the contacts between individuals that result in scabies infestation, do not occur at random [26]. Mixing patterns are a characteristic of a network (e.g. community) referring to the extent to which nodes (e.g. people) connect (e.g. are in close enough contact to result in infection).

Bhunu et al. [23] published a deterministic, compartmental model, using Descartes’ rule of signs and numerical simulations to show endemic equilibria and determine whether the current treatment regime is sufficient to control scabies infection, or whether a vaccine is required. Assumed values for key model parameters were not substantiated, and it is not clear what the model is calibrated to. This means it is impossible to evaluate the reliability of the model and any of its results. The model focussed on predicting the potential impact scabies vaccination might have in case a vaccine would become available. It should likely be viewed as a theoretical exercise rather than one that provides actual insights into scabies epidemiology or into the effectiveness of any available intervention.

Lydeamore et al. [24] recently published another compartmental model to explore the impact of alternative MDA treatment strategies. As opposed to the other models, this model aimed to capture the mite’s life cycle in relation to the host. The authors considered this critical, as the parasite’s life state (e.g. eggs versus living mites) can interact critically with treatment success or failure. This is a valuable model characteristic when studying the effectiveness of different treatment types (e.g., ovicidal versus non-ovicidal). In contrast to the model by Gilmore at al., homogeneous mixing is assumed.

None of the models included QoL. An advantage of including QoL is that it can be used to quantify the impact of a wide range of conditions and that (under certain requirements) it can be multiplied with duration of life to obtain QALYs (quality-adjusted life years). By measuring the impact of interventions on QALYs, outcomes can be compared not only between different (types of) interventions but also across different disease areas. This is needed to inform decision-making, particularly when funds need to be distributed over interventions/programs in a range of different areas. In models, QoL can be used as outcome measure, for example by weighting health states (e.g. “CS grade 1”) by their associated utility value. Furthermore, disutilities can be attached to complications as well as treatment-related adverse events. The same is true for costs, which can be attached to the various health states and events in cost-effectiveness models.

Only the model by Bachewar et al. included a cost-effectiveness analysis, but it did not take into account transmission dynamics.

### Proposed scabies modelling framework

**Fig 2:**
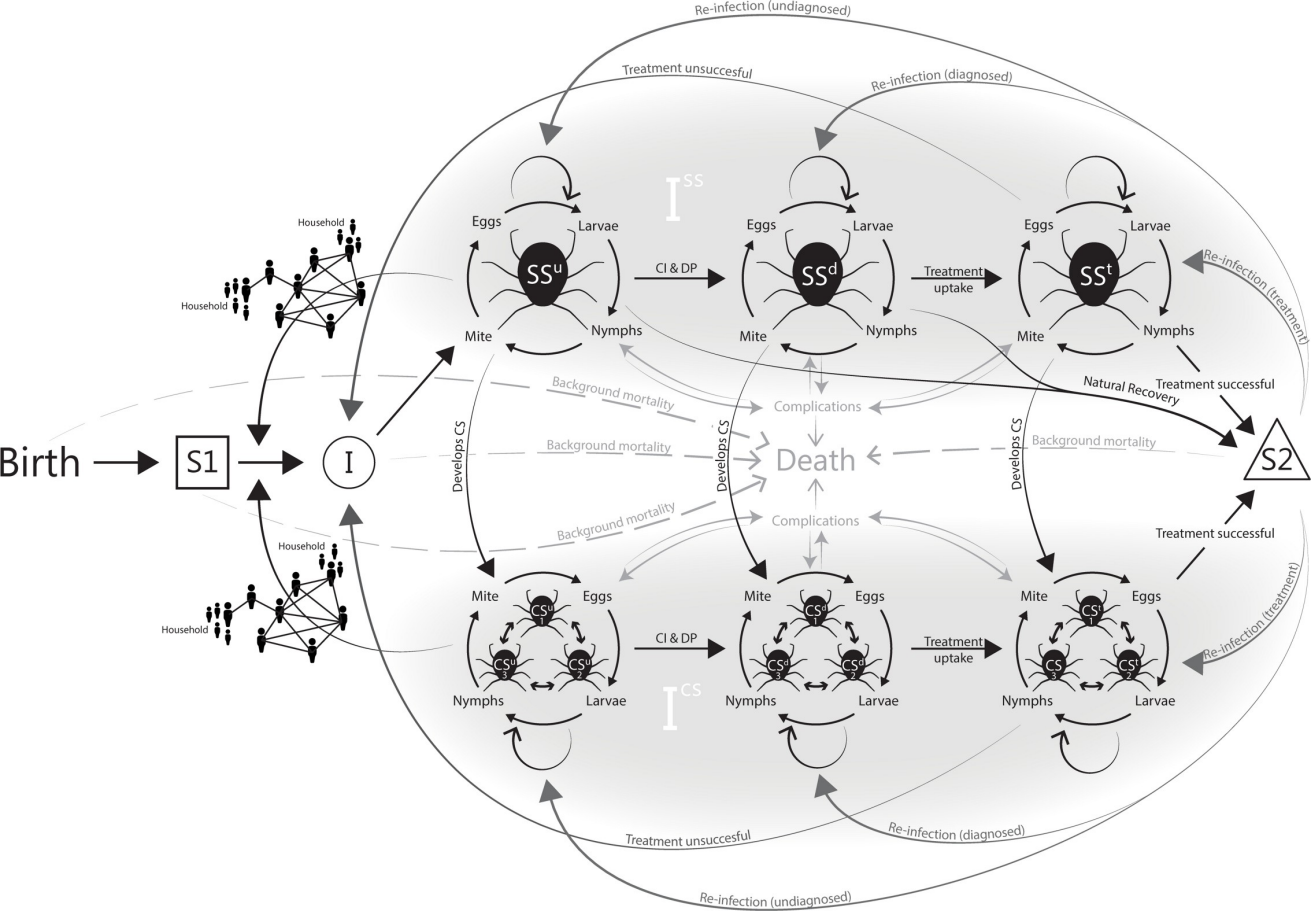
Scabies transmission model. Abbreviations: CI & DP, case identification and diagnostic processes; CSd, crusted scabies diagnosed; CSu, crusted scabies undiagnosed; CSt, crusted scabies treatment; I, infectious; R, recovered; S1, susceptible (never has been scabies infected); S2, susceptible (after prior scabies infection); SSd, simple scabies diagnosed; SSu, simple scabies undiagnosed; SSt, simple scabies treatment. The figure shows a susceptible population (S1) of individuals who can be infected (I) with scabies. The probability of infection depends on the interaction between individuals with other, infected, members of their household or community reflected by the network diagrams. Infected patients develop simple scabies (ISS), which is initially undiagnosed (SSu). Case identification and diagnostic processes (CI & DP) determine the probability that a patient becomes diagnosed (SSd). Subsequently, there is a probability that a patient takes up treatment and moves to SSt. Treatment has a probability of success, dependent on treatment efficacy and compliance. In this case, patients are cured and susceptible for reinfection (S2), the probability and infectiousness of which can differ from the probability and infectiousness in individuals who never had scabies before. Re-infection or sustained infection after unsuccessful treatment can go undiagnosed if a patient does not receive appropriate follow-up. A proportion of patients with simple scabies will develop CS. Grade 1 CS (CS1) can progress to grade 2 CS (CS2) and grade 3 CS (CS3). While both simple scabies and CS can result in complications, the probability of this is higher in patients with CS and increases with higher grade CS. Complications can result in death or can resolve. “Background mortality” reflects people who die from other causes than CS, which can differ between the susceptible and infected populations due to differences in comorbidities. In case the model is used to inform an economic evaluation, each of the health states (S1, SSu, SSd, SSt, CSu (grades 1–3), CSd (grades 1–3), CSt (grades 1–3) and S2) is associated with a cost and a utility. Diagnostics, treatments and complications can be associated with additional costs and/or (dis)utilities. The model can be used to simulate a community and how individuals move through the various health states over time, accruing costs, life-years and utility based on the time they spend in each of the health states. Results can be expressed in terms of costs per quality-adjusted life year (QALY) gained, or any other outcome captured in the model (e.g. cost per reinfection prevented or cost per life year gained).

Combining the strengths of the abovementioned models, Fig 2 provides a proposed modelling framework to inform cost-effectiveness analyses. This framework can be used as aid for creating a scabies transmission model, the details of which will be determined by the context (population) and the question being addressed. Like the model by Gilmore (2011), it allows for modelling networks and mixing patterns. This is a valuable attribute in case the model will be used for evaluating the cost-effectiveness of interventions which aim, for example, to prevent reinfection through household level interventions (e.g. ensuring treated CS patients return to scabies-free homes), or community interventions to reduce the prevalence of scabies. Mixing patterns can be based on assumptions or, preferably, appropriate data collection. For example, to inform scabies transmission modelling in indigenous communities in Australia, information is currently being collected on the number of infections and reinfections in the various communities, living conditions (e.g. number of persons per household), and the extent to which scabies-free zones are being established when CS patients return from the hospital. Network size and structure will be dependent on the type of community that is being modelled, including age structure since children tend to have a higher probability of acquiring scabies [27]. For more information on challenges when modelling contact networks, see Eames et al. 2015 [28].

The proposed modelling framework also aims to capture the biology and natural history of scabies transmission in humans, in particular the life-cycle of the mites as this can impact treatment success rates [24]. Depending on the modelling question at hand, it may be possible to simplify the proposed modelling framework or use other types of modelling methods. For example, when the difference between ovicidal versus non-ovicidal treatments is irrelevant to the question at hand, it may not be needed to track life-cycle stages of mites in individual patients. As a rule, simple models should be preferred over complex ones when the decision problem allows, since they are easier to understand, less prone to inaccuracies, and quicker to develop and run [29]. On the other hand, oversimplification may result in unreliable or invalid results when relevant risk-factors or dependencies between modelled states or agents are not taken into account. While it may be possible to simplify the model for some questions, others might require additional health states, for example to allow modelling of long-term complications (e.g. chronic renal failure) and their effects. For more information on different types of modelling methods, see Siettos and Russo 2013 [30].

Within the proposed modelling framework, model time should be counted in days, to account for processes like infection, the scabies life-cycle, and treatment effects. Other processes may take substantially longer, such as processes related to certain complications, and impacts on life expectancy. For most modelling questions, a life time horizon will be sufficient.

None of the identified models explicitly included CS. This is a shortcoming, since CS is associated with high infectivity, morbidity and mortality compared to simple scabies, and has often been overlooked in scabies program design. The proposed modelling framework incorporates a probability of moving from simple scabies to CS, which can be dependent on (amongst other factors) immune status of the patient.

While other infectious disease models often include some notion of seasonality, this has not been the case for scabies models. Although scabies mites show increased mite movement and increased transmission in a warm environment, a study in Malawi found that scabies was more prevalent during the cold, dry season, possibly due to close interpersonal contact in crowded indoor environments [31]. Other studies, however, show no obvious seasonal variation at all [31–33]. In the absence of evidence to the contrary, the proposed modelling framework does not accommodate seasonality.

The following sections of this paper discuss input parameters that can be used to inform the suggested modelling framework, or other newly developed scabies models.

### Life cycle of scabies mites

Following Lydeamore *et al*. [24], it is crucial to account for the life cycle of the scabies mites to model treatment effectiveness, especially when aiming to discriminate between ovicidal and non-ovicidal treatments. The literature review identified 14 articles presenting information on the life cycle of scabies mites (see Table 3). Since there is often a lack of *Sarcoptes scabiei* var. *hominis* mites, many studies have relied on animal strains of scabies mites and a host animal model such as rabbits or pigs [34]. The studies obtained through the search strategy did not provide any other information specifically on the life cycle of *Sarcoptes scabiei* var. *hominis* apart from “survival away from host”. However, based on direct comparisons, *Sarcoptes scabiei* var. *canis* seems to be a suitable model for *sarcoptes scabiei* var. *hominis* [34, 35].

**Table 3:** Life cycle of *Sarcoptes scabiei*.

*Sarcoptes scabiei* (var. not specified)			
Time it takes until pregnant mites begin tunnelling once transferred		1 hour	[36]
Number of eggs per mite		2–3 per day	[24, 36]
		2–4 per day over 4–6 weeks	[37]
		in total	[38, 39]
Egg incubation time		2–3 days	[40]
		2–4 days	[37]
		48 hours	[36]
		50–53 hours	[41]
Larval stage		3–4 days	[40, 41]
Nymphal stages		days	[40]
		10–13 days	[41]
Adult stage		1–2 months	[40]
Development from egg to adults / total life cycle		10–17 days	[36]
		10–15 days	[38]
		10–14 days	[42]
		12–17 days	[43]
		17–21 days	[44]
		7–10 days	[45]
		days	[46]
		~15 days	[39]
Percentage of eggs that develops into mature mites		<10%	[42]
Time it takes for an adult mite to find a mate		˜2 weeks	[24]
Time from initial infestation until second generation of adult mites appears		˜30 days	[24, 41]
Mortality of mites after hatching		90%	[36]
Survival away from host		Up to 3 days	[38]
Mean duration of life of the mite		30 days	[42]
		26–40 days	[39]
*Sarcoptes scabiei* var. *hominis*			
Survival away from host	24–36 hours (at room conditions)		[35, 36]
*Sarcoptes scabiei* var. *canis*			
Development from egg to adult / total life cycle		9.93–13.03 days for females, 10.06–13.16 days for males	[47]
Egg incubation time		50.1 (sd 2.45)– 52.97 (sd 3.26) hours	[47]
Larval stage		3.22 (sd 1.52)– 4.20 (sd 1.52) days	[47]
Protonymphal stage		2.40 (sd 0.84)– 3.40 (sd 0.84) days for females, 2.33 (sd 0.66)– 3.33 (sd 0.66) days for males	[47]
Larval and protonymphal stage combined		(sd 1.29)– 7.5 (sd 1.29) days	[47]
Tritonymphal stage		2.22 (sd 1.01)– 3.22 (sd 0.97) days for females, 2.42 (sd 0.51)– 3.42 days (sd 0.51) for males	[47]
Nymphal stages combined		2.35)– 6.67 (sd 2.35) days	[47]
Proportion of mites which die in the burrow		9%	[47]
Survival away from host		24–36 hours (at room conditions)	[35]

Abbreviations: NR, not reported; sd, standard deviation.

### Quality of life

The literature review identified 3 QoL studies performed in scabies patients: 1 from China (= 96), 1 from Brazil (n = 105) and 1 from India (n = 102). None of these studies addressed the QoL of CS patients compared to the QoL associated with simple scabies, and two of the studies [48, 49] excluded CS patients. Jin-Gang et al. [48] used the Dermatology Life Quality Index (DLQI), and Worth [50] and Nair [49] used a modified version of that same questionnaire. Modifications made by Worth et al. included: 1) adapting the language to local culture and attitudes; 2) modifying questions to increase relevance for persons living in an urban slum in the tropics; and 3) changing questions that were not applicable in children. Nair et al. used the modified version from Worth et al., with slight modifications as per the requirements of the Indian population.

Table 4 shows QoL results from the three studies, as per category of effect from scabies on QoL. Note that categorisation was based on classifiers described in the studies (e.g. “small effect”), not the modified DLQI item scores, since the questionnaires differed slightly between studies. Jin-Gang et al. reported a mean DLQI score of 10.09 (sd 5.96), with most QoL impact of scabies due to symptoms, embarrassment, work or study and sexual difficulties. Most common categories of impairment according to Worth et al. were feelings of shame (77.2% in adults, 46.6% in children), the need to dress differently (35.1% in adults, 29.3% in children), restriction on leisure activities (24.6% in adults, 36.8% in children), stigmatisation at work/school (21.1% in adults, 25.0% in children), social exclusion (24.6% in adults, 17.9% in children), teasing (26.3% in children), and problems with sexual partners (10.9% in adults). Women/girls perceived more restrictions than men/boys.

**Table 4:** Quality of life results.

	Worth et al. 2012	Nair et al. 2016	Jin-Gang et al. 2010	Worth et al. 2012	Nair et al. 2016
	Children		Adults		
**No effect on QoL**	˜22%^1^	62.5%	4.17%	˜19%^1^	24.2%
**Small/mild effect on QoL**	39.7%	27.5%	17.70%	28.1%	51.6%
**Moderate effect on QoL**	25.9%	10.0%	37.50%	36.8%	24.2%
**Large/very large effect on QoL**	˜12%^1^	0.0%	34.38%	˜16%^1^	0.0%
**Extremely large effect on QoL**	-	-	6.25%	-	-

Abbreviations: QoL, quality of life.

^1^Not reported: read from Figure.

A review of studies using the Children’s Dermatology Life Quality Index (CDLQI) questionnaire to measure QoL in skin conditions [51] found an overall estimated CDLQI score of 9.2 (95%CI: 0.0–20.3) associated with scabies. The review identified two studies that were not identified in our literature review: Balci et al. [52] and Lewis-Jones & Finlay [53]. Both included children with a wide range of skin diseases, including only few scabies patients (n = 9 and n = 6, respectively). Olsen et al. commented that while scabies might have a large effect on QoL at the time of completing the questionnaire, this may only be over a short time as it is curable. While also curable, the disutility of CS may be more substantial, given the severity of associated symptoms and complications.

### Resource use

While simple scabies is relatively straightforward to treat, patients may not seek care, may wait a long time before doing so, and may be misdiagnosed. In Cameroon, Kouotou et al. [54] found that it takes 4 to 720 days between the onset of symptoms and the first consultation with a dermatologist, with a mean of 77.1 days (sd 63.7). At the first consultation with a dermatologist, 74.9% had already tried previous treatment, such as antibiotics, antifungals, antihistamines or plant-based medicines.

Based on claims data for the employer-sponsored privately-insured population in the United States, treating one episode of scabies costs on average 95 USD [55]. When selecting on episodes for which drug treatment was claimed, costs were 163 USD per episode. Given the incidence of scabies, this results in an overall annual economic burden of 10.4 million USD for treating scabies in this population, most of which (3.7 million USD) is for children ≤15 years. Costs between alternative treatment options differ substantially. For a cost-benefit analysis from a US perspective, we refer to Elgart [56].

In some populations, scabies-associated health resource use is substantial. In five remote communities of Northern Australia, only a few aboriginal children (16%) manage to reach their first birthday without having at least one documented episode of scabies and/or skin sores [57]. Here, the median number of presentations per child under 12 months due to scabies is 3 (IQR 1, 5). Of these children, 70.5% present more than once, and the average age of first presentation is 4 months (IQR 2–7). In another Australian study, Whitehall et al. [32] found that the mean duration of hospital admissions for children with scabies is 4.5 days. Scabies comprised 4.2% of the total number of admissions for all reasons, and 8.3% of all bed days. The minimum cost per admission was 9,584.07 AUD. In Australia, the estimated annual cost associated with the management of pediatric scabies and pyoderma per patient was 10,000 AUD in 2013 [58].

Resource use may differ substantially between locations/communities, depending on the healthcare system, funding, remoteness, and cultural differences, amongst other factors. Local data collection will generally be required to inform model inputs. Resource use or costs for treating CS have not been published. A cost-of-illness study from an Australian perspective is currently being performed by (part of) the authors of this paper. Note that the cost-effectiveness of interventions to prevent CS may be substantially impacted by their ability to prevent long-term complications (e.g. rheumatic fever and chronic valvular heart disease) which may increase costs and decrease patient life expectancy and quality of life.

## Discussion

Based on a systematic literature review, this paper discusses published models and proposes a new, comprehensive modelling framework to develop cost-effectiveness analyses of treatments for scabies. Models should be informed by population, disease and treatment characteristics, which may differ between communities. Available information on required model inputs was systematically reviewed. Prior to this review, the literature lacked a good account of these inputs, including the life cycle of scabies mites, patient QoL, and resource use. This review resolves this problem and should be supplemented by locally specific data collections and expert opinion where required.

There is a lack of reliable, comprehensive information about scabies biology and the impacts this disease has on patients and society. This may be due to the limited amount of resources directed towards scabies research [7, 8], and its tendency to affect resource-poor populations. Given the efficacy of available treatments and the relatively low costs of these treatments (although still prohibitively expensive in some low-income settings), current high prevalence rates of scabies are unacceptable. Interventions should aim to reduce scabies incidence in a sustainable, cost-effective manner. In doing so, it may be worth focusing additional efforts on identifying and treating patients with CS, who can be “core transmitters” of the disease, while experiencing high morbidity and mortality rates [38]. The importance of targeting CS patients has often been overlooked in program design for simple scabies.

Scabies elimination efforts should be prioritised for communities that are worst affected, and with sustained intervention, this is a realistic goal [59]. Given that many of these communities are resource-poor, cost-effective use of resources is crucial and can be informed by health-economic modelling, taking into account community-specific resource constraints and expected budget impact of proposed interventions. Furthermore, careful data collection (for example, aided by making scabies a notifiable disease) may help guide funds to where they are most needed.

While the current article provides a comprehensive overview of key issues and a proposed modelling framework to aid future scabies modelling work, it is only a first step in this direction. Researchers and policy makers are encouraged to use and adjust this modelling framework to develop an economic evaluation predicting the (cost)-effectiveness of interventions against scabies in their population(s) of interest. Any input on the proposed modelling framework by external parties is welcomed.

As with all health economic models, model transparency and validation of the results is critical to its success and potential impact. The current modelling framework has not been validated and should only be used as an aid for model development. By using the current review and proposed modelling framework to substantiate the modelling approach and select appropriate inputs, transparency can be improved. Proper validation involves face validity, verification of internal validity, external validity, and predictive validity. Health economists and modellers working in the field of scabies are referred to the ISPOR report on model transparency and validation for recommendations on how to appropriately validate and report on their model and results in a population of interest [60].

For data inputs that are uncertain, real-world data collection may be crucial to ensure reliability of modelling outcomes. Furthermore, the impact of uncertain model inputs can be tested by using sensitivity analyses to determine how variation in modelling inputs impacts the results, both deterministically and probabilistically. Given the identified knowledge gaps, it is important to perform extensive sensitivity analysis in any scabies model that will be developed. Meanwhile, grant bodies are encouraged to invest in scabies research to address the knowledge gaps identified in this review regarding the biology, QoL and cost impact of simple scabies and CS.

As far as the authors are aware, transmission modelling has seldom been used to answer questions on scabies interventions. One reason for this may be the lack of readily available information to inform modelling work, which this review aims to (at least partially) address. Another reason may be unfamiliarity or scepticism on the side of authorities and funding bodies with respect to the value of theoretical results obtained from modelling. Health economists and other scientists can best illustrate the value of modelling by using evidence-based, validated approaches to tackle relevant, real-world questions which can directly inform clinical or governmental decision-making.

## Supporting information

S1 Checklist PRISMA checklist. (DOC)
